# Absence of a Written Employment Contract and Health Outcomes Among Employed Adults in Chile

**DOI:** 10.3390/ijerph23030360

**Published:** 2026-03-12

**Authors:** Gonzalo Bravo-Rojas, Maythe Páez-Guajardo, Carlos Viviani, Ignacio Castellucci

**Affiliations:** 1 Escuela de Kinesiología, Facultad de Salud, Universidad Santo Tomás, Viña del Mar 2520000, Chile; 2 Centro de Estudio del Trabajo y Factores Humanos, Escuela de Kinesiología, Facultad de Medicina, Universidad de Valparaíso, Valparaíso 2340000, Chile; maythe.paez@postgrado.uv.cl; 3 Escuela de Kinesiología, Facultad de Ciencias, Pontificia Universidad Católica de Valparaíso, Valparaíso 2340000, Chile

**Keywords:** informal employment, precarious employment, labor contract, mental health, quality of life, Chile, social determinants of health

## Abstract

**Highlights:**

**Public health relevance—How does this work relate to a public health issue?**

- Informal employment remains widespread in Latin America and represents a structural determinant of health and social inequality.
- This study examines whether the absence of a written employment contract, as a proxy of labor informality, is associated with health and well-being outcomes among employed adults in Chile.

**Public health significance—Why is this work of significance to public health?**

- Using nationally representative survey data from Chile (ENSEX 2022–2023), the study provides updated evidence on the relationship between contractual informality and multiple health indicators.
- The findings show that workers without a written contract have higher odds of anxiety and depressive symptoms and lower quality of life, highlighting the relevance of employment conditions for psychological well-being.

**Public health implications—What are the key implications or messages for practitioners, policy makers and/or researchers in public health?**

- Policies aimed at improving workers’ health should consider employment quality and informality as key social determinants of well-being.
- Strengthening labor formalization and expanding occupational health strategies to include workers in informal employment may contribute to reducing health inequalities.

**Abstract:**

Precarious and informal employment has been increasingly recognized as a key social determinant of health, particularly in countries of the Global South. In Chile, despite relatively strong labor institutions, informal employment remains widespread, yet contemporary evidence on its health implications is limited. This study examines the association between the absence of a written employment contract, used as an indicator of labor informality, and multiple health and well-being outcomes among employed adults in Chile. A cross-sectional analysis was conducted using data from the nationally representative 2022–2023 National Health and Sexuality Survey (ENSEX), restricted to the urban employed population (*n* = 5193). Informal employment was defined by self-reported absence of a written contract. Health outcomes included perceived general health, quality of life, physician-diagnosed conditions, and recent anxiety–depressive symptoms assessed with the PHQ-4. Weighted descriptive analyses and logistic regression models were estimated, accounting for the complex survey design and adjusting for sex, age, and educational level. Approximately 12.8% of employed individuals reported not having a written contract. Contract absence was associated with higher odds of anxiety–depressive symptoms and lower odds of reporting good quality of life after adjustment. Associations with general health and chronic physical conditions were weaker and not statistically significant. These findings suggest that contractual informality is particularly linked to reduced psychological well-being and quality of life, highlighting the relevance of informal employment as a public health concern beyond traditional disease outcomes.

## 1. Introduction

Precarious employment has emerged as a growing social determinant of health and an expanding phenomenon in contemporary labor markets. According to the systematic review by Kreshpaj et al. [1], which synthesizes how precarious employment has been conceptualized in empirical research, precarious employment should be understood as a multidimensional construct encompassing three core dimensions: job insecurity, reflected in contractual instability, temporary contracts, underemployment, and fragmented employment trajectories; income inadequacy, expressed through low wages, insufficient or unstable earnings, and the absence of economic benefits; and a lack of rights and protections, including limited social protection, weak labor regulation, low unionization, and constraints on exercising basic labor rights. These dimensions do not operate in isolation but tend to accumulate, shaping employment conditions marked by structural vulnerability and limited protection. Importantly, precarious employment is not defined by its physical or mental health consequences, but by the quality of the employment relationship and the institutional conditions that sustain it. Within this framework, understanding precarious employment as a complex and multidimensional phenomenon is essential for adequately assessing its implications for health and social inequalities [1].

This multidimensional framework also helps to elucidate related labor phenomena, including informal employment, which, although not synonymous with precarious employment, incorporates several of its core elements [2]. According to the International Labour Organization (ILO), informal employment refers to work arrangements that, in practice or by law, are not subject to national labor legislation, income taxation, or access to social protection or other labor guarantees, including the absence of advance notice of dismissal, severance pay, or paid leave [3]. The ILO further notes that informality may also occur within the formal sector, for example, among temporary, casual, or seasonal workers who lack social protection coverage or access to labor benefits. Within this context, the absence of a written employment contract constitutes a frequent manifestation of labor informality and exposes workers to heightened vulnerability by placing them outside institutional mechanisms of regulation, oversight, and social security [4]. Although precarious employment requires multidimensional approaches, the absence of a written contract remains a key indicator of vulnerability, closely linked to access to labor rights and social protection, and therefore relevant for public health research [5].

A substantial body of evidence has documented that precarious employment constitutes an important social determinant of health. Rönnblad et al. [5], in a systematic review and meta-analysis of longitudinal studies, demonstrated that precarious employment conditions are consistently associated with poorer mental health outcomes. In their analysis, job insecurity showed a sustained adverse effect on indicators of psychological distress (pooled OR = 1.52; 95% CI: 1.35–1.70). Importantly, the same review showed that multidimensional exposures to precariousness, integrating contractual instability, income inadequacy, lack of social protection, and restricted labor rights, exhibited even stronger associations with adverse mental health outcomes (OR = 2.01) [5]. Complementing this evidence, a recent systematic review reported that higher levels of precarious employment are associated with widespread deterioration in health and well-being, including poorer general health, greater functional limitations, increased job-related stress, and lower emotional well-being, with a clear gradient according to the degree of precariousness [6]. Similarly, Pulford et al. [7], focusing predominantly on longitudinal studies, found that exposure to precarious employment for 12 months or longer was associated with a 53% higher likelihood of reporting poor general health and a 44% increase in poor mental health symptoms. Taken together, this body of evidence indicates that precarious employment affects multiple dimensions of health and well-being, underscoring the relevance of employment quality as a central public health concern [7].

However, despite the robustness of the international evidence, quantitative research on labor precariousness and informal employment in Chile remains limited. Notably, the few available studies are based on data from the 2009–2010 National Survey of Employment, Work, and Health Conditions (ENETS) [8,9,10], a dataset that has not been updated for more than a decade. As a result, current knowledge on the relationship between informal or precarious employment and health in Chile relies on evidence that predates substantial social, economic, and labor market changes. This persistent reliance on outdated data underscores the lack of recent, nationally representative, and methodologically robust analyses addressing informal and precarious employment in the contemporary Chilean labor context.

Within the broader context of the Global South, informal employment constitutes a central mechanism through which social and health inequalities are reproduced. In many middle- and low-income countries, informality remains a dominant form of labor organization, characterized by unstable employment relations, limited access to social protection, and weak institutional regulation, with well-documented implications for health and well-being [11]. In Latin America, and particularly in Chile, informal employment has been shown to disproportionately affect socially and economically vulnerable groups, reinforcing existing inequalities in working conditions and health outcomes [12]. Although Chile exhibits comparatively favorable macroeconomic indicators and a relatively formalized labor market, informal employment continues to affect a substantial proportion of the workforce. Understanding informal employment as a structural feature of labor markets in the Global South is therefore essential for interpreting its health consequences and for situating the Chilean case within broader international debates.

In Chile, informal employment remains a widespread and structural form of labor market participation. According to the Informal Employment Bulletin of the National Institute of Statistics (July–September 2025), 26.2% of the employed population works under informal conditions, equivalent to more than 2.4 million people [13]. Informality affects population groups unequally: it reaches 28.1% among women and 24.8% among men and presents its highest levels among workers aged 65 and older (57.6%) and youth aged 15 to 24 (37.4%). Likewise, the highest rates are concentrated among self-employed workers (64.6%) and in traditionally vulnerable occupations such as agriculture, fishing, crafts, trades, and domestic work. These data indicate that informality is not only extensive but also deeply segmented by sex, age, economic sector, and occupational category, shaping a labor landscape marked by limited social protection and disproportionate vulnerability, with potential implications for health and well-being [13].

Given this context, it becomes necessary to examine whether the absence of a written employment contract is associated with poorer health and well-being outcomes among employed individuals in Chile. Understanding this relationship is relevant for informing public policies related to occupational health, employment regulation, social protection, and preventive strategies in the workplace. Within this framework, the present study aims to analyze the association between precarious and informal employment, operationalized through the absence of a written contract, and multiple health indicators among the employed population in Chile. Specifically, the study seeks to: (i) describe the distribution of health indicators according to contractual status; (ii) estimate the crude association between contract absence and different health outcomes; and (iii) assess this relationship after adjusting for relevant sociodemographic variables, including sex, age, and educational level.

## 2. Materials and Methods

### 2.1. Study Design and Data Source

This study used a cross-sectional design based on data from the 2022–2023 National Health and Sexuality Survey (Encuesta Nacional de Salud y Sexualidad, ENSEX 2022–2023), a nationally representative survey of the urban population in Chile [14]. Data were collected through face-to-face interviews administered by trained interviewers to adults residing in private households. All interviews were conducted in Spanish, which is the official language of Chile. ENSEX gathers information on health status, health-related behaviors, well-being, and sociodemographic characteristics. The survey employs a probabilistic, stratified, and multistage sampling design, allowing for population-level inference.

### 2.2. Study Population

From the original ENSEX 2022–2023 sample, all individuals aged 18 years or older who reported being employed at the time of the survey (i.e., having a current job or economic activity, according to the survey definition) and who had complete information on contractual status and the health indicators examined were included in the analysis. The analytical sample was restricted to wage employees, as the exposure variable required the presence or absence of a signed written labor contract in the respondent’s main job. Self-employed individuals were therefore not included in the analysis. The final analytical sample consisted of 5193 employed individuals.

### 2.3. Variables of Study

#### 2.3.1. Main Exposure: Informal Employment

The exposure of interest was defined based on the presence or absence of a written employment contract, which was used as an indicator of informal employment. This decision was grounded in the internationally accepted definition of informal work, particularly as formulated by the International Labour Organization (ILO). According to the ILO, informality is characterized by the absence of legal coverage or insufficient regulatory protection of employment, which explicitly includes the lack of a written employment contract [3].

Accordingly, this variable was constructed using question p280 of the ENSEX 2022–2023 survey (“In your main job, do you have a written employment contract?”). Response options included: “Yes, signed,” “Yes, but not yet signed,” “Does not have one,” “Does not remember/does not know whether they signed a contract,” and “No response.” A dichotomous variable was created by classifying respondents as “with contract” if they answered “Yes, signed,” and as “without contract” if they answered “Does not have one,” which was interpreted as the absence of contractual formality. To minimize misclassification, responses indicating a contract in process (“Yes, but not yet signed”), ambiguity (“Does not remember/does not know whether they signed a contract”), or missing data (“No response”) were excluded from the analysis. The resulting variable was used both in the descriptive analyses and in the estimation of associations with health outcomes. These exclusions resulted in the final analytical sample used in all descriptive and regression analyses.

#### 2.3.2. Health Indicators (Outcome Variables)

General health: Perceived general health was assessed using the question, “In general, how would you rate your health?”, and dichotomized into good or very good versus fair, poor, or very poor.

Depression and anxiety–depressive symptoms: Physician-diagnosed depression was assessed using the question, “Has a physician ever told you that you have or suffer from depression?” (yes/no). Additionally, recent psychological symptomatology was measured using the abbreviated Patient Health Questionnaire-4 (PHQ-4), which assesses symptoms of anxiety and depression over the previous two weeks [15]. For this study, a dichotomous variable was constructed distinguishing individuals without symptoms (PHQ-4 = no symptoms) from those with anxiety–depressive symptomatology, grouping mild, moderate, and severe categories. Responses of “Don’t know/No response” were excluded from the analysis. This measure captures current psychological distress, complementing estimates based on professional diagnoses.

Hypertension: Hypertension was identified through the question, “Has a physician ever indicated that you have high blood pressure or hypertension?” (yes/no).

Overweight or obesity: Overweight or obesity was assessed using the question, “Has a physician ever told you that you have overweight or obesity?” (yes/no).

Quality of life: Quality of life was evaluated using the question, “In general, how would you rate your quality of life?”, and dichotomized into good versus poor.

Other physician-diagnosed conditions: Chronic musculoskeletal disorders were identified through the question, “Has a physician ever diagnosed you with a chronic musculoskeletal disorder, such as persistent pain in the back, neck, or extremities?” (yes/no). Infertility was determined using the question, “Has a physician ever told you that you have infertility or permanent difficulties conceiving?” (yes/no), and hepatitis B was assessed through the question, “Has a physician ever diagnosed you with hepatitis B?” (yes/no).

All health and well-being indicators were described for the total population and analyzed according to contractual status (with versus without a written employment contract).

#### 2.3.3. Covariables

The adjusted models included the following sociodemographic covariates, which were selected a priori based on their theoretical and empirical relevance in the relationship between employment conditions and health outcomes:

Sex: Sex was included as a binary variable (male/female).

Age: Age was treated as a continuous variable in regression models. For descriptive purposes, age was categorized into four groups: 18–29 years, 30–44 years, 45–64 years, and 65 years or older.

Educational level: Educational attainment was classified into five categories: early education, primary education, secondary education, technical–vocational education, and professional or postgraduate education.

### 2.4. Statistical Analysis

Given the complex sampling design of ENSEX 2022–2023, all analyses incorporated the survey stratification, primary sampling units, and expansion weights. The survey design was specified using the svyset command, and all estimations were conducted using svy prefixes. All analyses were performed using Stata version 16 (StataCorp LLC, College Station, TX, USA).

First, a sociodemographic characterization of the employed population was performed. Weighted proportions and their corresponding 95% confidence intervals (95% CI) were estimated for sex, age groups, educational level, contractual status, and health indicators using svy: proportion. To compare the distribution of variables according to contractual status, weighted contingency tables were generated using svy: tab. Group percentages and the design-based Wald statistic (F), reported as χ^2^ (Wald), were presented together with the corresponding *p*-values.

Second, the association between the absence of a written employment contract and each health indicator was assessed using weighted logistic regression models, which were fitted independently for each outcome. In all models, individuals with a written employment contract were used as the reference category. Two types of models were estimated: (i) crude models including only contractual status, and (ii) adjusted models incorporating sex, age (continuous), and educational level. Results were expressed as odds ratios (ORs) with their corresponding 95% CI and *p*-values.

A significance level of *p* < 0.05 was applied for all two-sided tests. In the presence of missing data for variables of interest, a complete-case approach was applied, restricting analyses to observations with available information on the exposure, covariates, and the corresponding outcome.

### 2.5. Ethical Considerations

The ENSEX 2022–2023 survey received approval from a scientific ethics committee and was conducted in accordance with national and international standards for research involving human participants, including the requirement to obtain informed consent from all respondents. The present study used anonymized secondary data, which prevented the identification of participants and eliminated the need for direct contact with them. The study was conducted and reported in accordance with the Strengthening the Reporting of Observational Studies in Epidemiology (STROBE) guidelines for observational studies [16].

## 3. Results

### 3.1. Sample Characteristics

The analysis included 5193 employed individuals who participated in ENSEX 2022–2023. As shown in Table 1, 60.2% of the sample were men and 39.8% were women. Regarding educational attainment, the largest proportion had completed secondary education (37.9%), followed by professional or postgraduate education (33.9%) and technical or vocational education (21.1%).

In terms of contractual status, 87.2% of participants reported having a signed written employment contract (95% CI: 85.3–88.8), while 12.8% reported not having a contract at the time of the survey (95% CI: 11.2–14.7).

### 3.2. Prevalence of Health Indicators

Overall, most participants reported a positive perception of their general health. Specifically, 74.6% rated their health as good or very good (95% CI: 72.5–76.6). Similarly, 80.3% reported a positive evaluation of their quality of life (95% CI: 78.3–82.1). Regarding mental health, 15.9% of participants indicated having received a professional diagnosis of depression at some point in their lives (95% CI: 14.2–17.9).

The prevalence of chronic diseases varied across conditions. Diabetes was reported by 7.1% of participants (95% CI: 6.0–8.4), while hypertension was reported by 12.0% (95% CI: 10.7–13.5). In addition, 25.3% of participants reported having been diagnosed with overweight or obesity (95% CI: 23.0–27.8).

Regarding musculoskeletal health, 13.8% of participants reported a diagnosis of chronic musculoskeletal disorders, including persistent pain in the back, neck, or extremities (95% CI: 12.3–15.5). Low-prevalence conditions were less frequently reported, with infertility reported by 1.5% of participants (95% CI: 1.1–1.9) and hepatitis B by 0.7% (95% CI: 0.4–1.2).

### 3.3. Association Between Absence of a Labor Contract and Health Indicators

Table 2 summarizes the crude and adjusted odds ratios (ORs) for the association between the absence of a labor contract and the different health indicators. In the crude model, individuals without a signed contract had lower odds of reporting good or very good general health compared with those with a contract (OR = 0.66; 95% CI: 0.48–0.90; *p* = 0.009). After adjustment for sex, age, and educational level, this association was attenuated and no longer statistically significant (OR = 0.78; 95% CI: 0.56–1.08; *p* = 0.139).

No statistically significant associations were observed between the absence of a contract and having received a physician diagnosis of depression, either in the crude model (OR = 1.31; 95% CI: 0.93–1.84; *p* = 0.119) or in the adjusted model (OR = 1.22; 95% CI: 0.86–1.74; *p* = 0.267). In contrast, a consistent association was found for anxiety and depressive symptoms assessed with the PHQ-4 scale. Individuals without a contract showed higher odds of reporting at least one symptom in both the crude (OR = 1.62; 95% CI: 1.28–2.07; *p* < 0.001) and adjusted models (OR = 1.59; 95% CI: 1.26–2.01; *p* < 0.001).

The absence of a labor contract was not significantly associated with chronic disease diagnoses. No associations were observed for diabetes in either the crude (OR = 0.87; 95% CI: 0.54–1.40; *p* = 0.575) or adjusted models (OR = 0.62; 95% CI: 0.32–1.17; *p* = 0.138), nor for hypertension (crude OR = 1.26; 95% CI: 0.90–1.76; *p* = 0.184; adjusted OR = 1.00; 95% CI: 0.61–1.63; *p* = 0.996). Similarly, no significant associations were found for overweight or obesity in the crude (OR = 1.05; 95% CI: 0.80–1.40; *p* = 0.707) or adjusted models (OR = 1.07; 95% CI: 0.82–1.41; *p* = 0.616).

Regarding quality of life, individuals without a contract had lower odds of reporting a good quality of life in the crude model (OR = 0.58; 95% CI: 0.43–0.79; *p* < 0.001). This association remained statistically significant after adjustment, although with reduced magnitude (OR = 0.71; 95% CI: 0.51–0.99; *p* = 0.042).

No statistically significant associations were observed between contract absence and musculoskeletal disorders in either the crude (OR = 1.14; 95% CI: 0.82–1.60; *p* = 0.437) or adjusted models (OR = 1.00; 95% CI: 0.72–1.40; *p* = 0.989). Likewise, infertility and hepatitis B showed no significant associations with contract status in any model, with wide confidence intervals reflecting the low prevalence of these conditions.

## 4. Discussion

This study examined the association between the absence of a written labor contract and a range of health and well-being indicators among employed individuals in urban Chile. The results show that employment without a contract was consistently associated with poorer quality of life and with higher odds of recent anxiety and or depressive symptoms, as measured by the PHQ-4, even after adjustment for key sociodemographic characteristics. In contrast, the absence of a contract was not independently associated with having received a medical diagnosis of depression, nor with most physical health indicators, including chronic diseases and musculoskeletal disorders. For self-rated general health, an association was observed in crude analyses, but this relationship attenuated and lost statistical significance after adjustment. Taken together, these findings indicate that contractual informality is more strongly related to subjective well-being and recent psychological distress than to diagnosed mental disorders or physical health conditions.

### 4.1. The Absence of a Contract as a Form of Informality and Labor Precariousness

In Chile, a series of regulatory reforms implemented over recent decades, including the expansion of unemployment insurance coverage [17], the introduction of mandatory pension contributions for self-employed workers [18,19], and the strengthening of labor regulation and enforcement [20,21], have aimed to reduce gaps in social protection and labor informality. Despite these efforts, the present study shows that nearly 13% of employed individuals in urban areas report not having a written labor contract, indicating that contractual informality remains a persistent feature of the Chilean labor market.

This proportion is lower than the 26% reported by the National Institute of Statistics [13], a difference that may be partly explained by the exclusion of rural and hard-to-reach areas from the survey sample. Nevertheless, the observed prevalence is consistent with regional evidence positioning Chile and Uruguay among the countries with the lowest rates of labor informality in Latin America [22]. Importantly, the ENSEX survey was conducted in urban settings where institutional presence and enforcement capacity are presumed to be stronger, suggesting that employment without a contract is not limited to contexts of weak regulation but extends across diverse productive sectors.

Beyond its descriptive relevance, this finding contributes to a field that remains relatively underexplored in Chile. Previous research has highlighted that, despite the magnitude of labor informality and precarization, empirical studies examining their health implications are still limited, in part due to the absence of standardized definitions and methodological challenges in measuring precarious employment conditions [9]. While studies using multidimensional measures of labor precariousness have documented associations with poorer general and mental health, as well as increased occupational injury risk among Chilean workers [10], and other research has linked informal employment to adverse physical and mental health outcomes [9,12], evidence specifically addressing contractual informality and its relationship with health indicators remains scarce. In this context, the present study provides recent empirical evidence supporting the relevance of contractual status as a meaningful dimension through which social, economic, and health-related vulnerabilities are structured in the Chilean labor market.

### 4.2. Impact of Contractual Informality on Health Indicators

Our findings indicate that the absence of a labor contract is associated with lower odds of reporting good or very good health in crude models; however, this association attenuates and loses statistical significance after adjustment for sociodemographic and occupational characteristics. From a compatibility-based interpretive perspective, these results are consistent both with a small adverse effect of contract absence on perceived health and with the absence of an independent effect, given the width of the confidence interval and the assumptions of the model. This pattern aligns with international evidence showing that contract type constitutes a partial indicator of labor precariousness and rarely retains independent associations with health outcomes once broader structural factors are considered.

In contrast, the association between the absence of a labor contract and poorer quality of life persists in both crude and adjusted models, although with reduced magnitude after adjustment. The compatibility of the confidence interval with a negative effect suggests that contractual informality represents a relevant vulnerability factor for quality of life, independent of basic sociodemographic characteristics. Compared with other health indicators, quality of life emerges as a particularly sensitive outcome, showing greater stability of association with contract absence when structural covariates are taken into account.

This finding may be related to the fact that a formal labor contract functions not only as a legal arrangement, but also as a gateway to employment stability, income predictability, labor rights, and social protection. These dimensions have been described as central mechanisms through which formal employment reduces uncertainty, material deprivation, and chronic stress, thereby influencing overall well-being [23]. Consistent with this interpretation, studies using multidimensional measures of labor precariousness have shown that domains related to vulnerability, insecurity, and restricted rights are robustly associated with poorer subjective well-being, including reduced autonomy, perceived control, and life satisfaction [6,24].

An important distinction emerges when comparing the two mental health indicators analyzed. The absence of a labor contract was not significantly associated with physician-diagnosed depression, although odds ratios remained above one. This lack of association may reflect the cumulative nature of diagnostic indicators and their dependence on effective access to health services, which can be unevenly distributed among workers in precarious employment situations [23].

In contrast, recent anxiety and/or depressive symptomatology measured using the PHQ-4 showed a robust and consistent association with contract absence, even after adjustment. This pattern aligns with evidence suggesting that labor precariousness operates as a psychosocial stressor that manifests initially through emotional distress and subclinical symptoms, prior to the development or diagnosis of mental disorders [5,25,26].

These findings are consistent with recent evidence from Latin American and Iberoamerican contexts. In a pooled analysis of 180,260 workers from 13 Iberoamerican countries, reported that informal employment was associated with a higher prevalence of poor mental health, with overall adjusted prevalence ratios of 1.19 among men and 1.11 among women [27]. Similarly, Huynh et al. (2022), analyzing data from 11 Latin American cities, found that workers in informal employment exhibited significantly higher levels of depressive symptoms compared to their formal counterparts, although the magnitude of the association varied across settings [28]. Importantly, both studies highlight heterogeneity between countries and suggest that the association is more consistent for subjective psychological outcomes than for clinically diagnosed conditions. In this regard, our results, which show a robust association with recent anxiety and depressive symptoms as well as with quality of life, but not with physician-diagnosed depression or most physical health indicators, are consistent with regional evidence indicating that employment informality primarily affects psychological well-being rather than established disease.

No statistically significant associations were observed between contract absence and physical health indicators, including chronic diseases, musculoskeletal disorders, and low-prevalence conditions. From a compatibility perspective, the estimates were consistent with small or null effects, with confidence intervals remaining close to one in both crude and adjusted models. This pattern is consistent with literature indicating that simple indicators of precariousness, such as contract type, have limited capacity to capture the structural mechanisms influencing physical health outcomes, which are more strongly shaped by cumulative exposures, physical work demands, and long-term etiological processes [6,24]. Taken together, these findings reinforce the interpretation that contract absence functions as a partial marker of labor disadvantage, whose health impact depends on broader dimensions of precariousness and on the specific characteristics of work.

This pattern suggests that simple contractual indicators are particularly informative for outcomes related to subjective well-being and recent psychological distress, while being less sensitive to conditions driven by long-term cumulative or biomedical processes.

### 4.3. The Absence of a Contract as an Indicator of Social Exclusion

From a broader perspective, the absence of a formal labor contract can be understood as an indicator of social exclusion, insofar as it reflects restricted access to basic labor rights, social protection, and mechanisms of economic citizenship. As described by Benach et al. [23], precarious and informal forms of employment are characterized by limited access to social security benefits, insufficient labor rights, and weak protection against major social risks, placing workers at the margins of institutional systems designed to ensure economic and social security.

In this sense, contractual informality does not merely represent a specific employment arrangement but rather constitutes a structural condition that constrains social integration and limits the full exercise of social and economic rights. The lack of a written contract often entails reduced access to health insurance, unemployment protection, and collective bargaining mechanisms, reinforcing situations of material deprivation, insecurity, and vulnerability that are central to processes of social exclusion.

Interpreting contract absence through the lens of social exclusion helps situate the observed associations with quality of life and psychological well-being within a broader framework of inequality. Rather than operating solely through direct effects on health, contractual informality may shape well-being by limiting access to resources, protections, and opportunities that support stable living conditions and social participation. From this perspective, employment without a contract reflects not only labor market disadvantage, but also a form of institutional exclusion with potential implications for health, well-being, and social equity.

### 4.4. Implications for Public Policy

The findings of this study have relevant implications for labor and public health policy in Chile. The persistent association between the absence of a labor contract and poorer quality of life, as well as the robust association with recent anxiety and or depressive symptoms, highlight that the health consequences of contractual informality extend beyond traditional biomedical outcomes. These results suggest the need to broaden the scope of occupational health and labor policies to explicitly incorporate psychological well-being and quality of life as central dimensions of workers’ health.

From a labor policy perspective, the results reinforce the importance of strengthening strategies aimed at employment formalization, while also recognizing that labor precariousness cannot be addressed solely through contractual status. Policies should therefore target broader dimensions of employment insecurity, including unstable working hours, subcontracting arrangements, low wages, and limited access to social protection, which may continue to affect workers’ well-being even in formally regulated labor markets.

In addition, the findings underscore the need to develop occupational health strategies that explicitly consider workers in informal or non-standard employment situations. This includes the implementation of preventive and educational interventions adapted to contexts where traditional employer-based occupational health services may be absent, as well as mechanisms to ensure access to basic health protection and psychosocial support for workers outside formal employment arrangements.

More broadly, addressing the health implications of contractual informality requires integrated policy approaches that combine labor regulation, social protection, and public health interventions. Strengthening enforcement mechanisms, expanding access to social security, and improving working conditions across diverse forms of employment may contribute to reducing inequalities in well-being and promoting healthier and more inclusive labor markets.

### 4.5. Strengths and Limitations

A major strength of this study is the use of a large sample drawn from a nationally representative survey, restricted to the urban employed population, which allows for robust and generalizable estimates of the association between contractual informality and health-related outcomes. The application of the survey’s complex sampling design, including stratification, primary sampling units, and expansion weights, enhances the inferential validity of the findings and reduces bias commonly present in non-probabilistic studies. In addition, the simultaneous assessment of multiple indicators of physical health, mental health, and well-being provides a comprehensive perspective on the potential health implications of employment without a labor contract.

Another strength lies in the use of adjusted analytical models that account for key sociodemographic characteristics, allowing for a clearer distinction between crude associations and those that persist after controlling potential confounding factors. By focusing on contractual status as a specific dimension of labor precariousness, this study also contributes novel evidence to a relatively underexplored area of occupational health research in Chile and the Latin American context.

Several limitations should be acknowledged. First, the cross-sectional design precludes establishing causal relationships or determining the directionality of the observed associations. Second, some health indicators were based on self-reported information, which may be subject to recall bias or social desirability bias, particularly for mental health diagnoses and chronic conditions. Third, contractual status was used as a proxy for labor precariousness and does not capture other relevant dimensions such as income instability, job insecurity, vulnerability, or exposure to psychosocial and physical work-related risks, potentially underestimating the complexity of precarious employment. Fourth, the ENSSEX survey was designed primarily to assess sexual health, not labor market conditions. Although it includes basic occupational variables that were incorporated into the analysis, it does not collect detailed information on economic sector or industry of employment. Therefore, we were unable to examine whether the association between absence of a written contract and health outcomes varies across specific occupational or sectoral contexts where informal employment may be more prevalent. In addition, employment information was collected only with respect to the respondent’s main job, making it impossible to determine whether participants held multiple jobs or to assess the characteristics of secondary employment. The survey also does not include detailed measures of job insecurity, psychosocial risk exposure, job control, rotating shifts, company size, wages, working hours, or job tenure, which may act as relevant mediating or confounding factors in the relationship between contractual informality and health outcomes.

Furthermore, the low prevalence of some health outcomes resulted in wide confidence intervals, limiting the precision of certain estimates. Although the models included key sociodemographic covariates, residual confounding related to unmeasured factors, such as specific job characteristics, working conditions, organizational support, or income level, cannot be ruled out. Despite these limitations, the findings provide meaningful evidence on the relationship between contractual informality and well-being, highlighting the importance of considering employment conditions as part of broader efforts to address health inequalities in the working population.

## 5. Conclusions

This study provides updated, nationally representative evidence on the relationship between the absence of a written labor contract and health and well-being among employed adults in urban Chile. The findings show that contractual informality is consistently associated with poorer quality of life and with higher levels of recent anxiety and depressive symptoms, even after accounting for key sociodemographic characteristics. In contrast, no independent associations were observed with physician-diagnosed depression or with most physical health indicators, and the association with self-rated general health attenuated after adjustment.

Taken together, these results suggest that the absence of a labor contract is more strongly linked to subjective well-being and recent psychological distress than to diagnosed mental disorders or physical health conditions. This pattern supports the interpretation of contractual informality as a partial marker of broader structural vulnerability rather than as a direct determinant of disease. From a public health perspective, the findings highlight the importance of incorporating indicators of employment quality, psychological well-being, and quality of life into research, surveillance, and policy discussions on labor conditions. Addressing the health implications of informal employment therefore requires approaches that go beyond biomedical outcomes and consider the broader social and institutional contexts shaping workers’ well-being. Although this study focuses on urban Chile, these findings are likely relevant to other contexts where informal or non-standard employment persists despite relatively strong labor institutions.

Future research should employ longitudinal designs to clarify the temporal direction of these associations and explore additional dimensions of labor precariousness beyond contractual status, including income instability, job insecurity, and working conditions. Further studies in Latin American contexts are also needed to better understand the structural mechanisms linking informal employment to health inequalities.

## Figures and Tables

**Table 1:** Sociodemographic characteristics and health indicators by contractual status (*n* = 5193).

Health Indicator	With Contract *n* (%)	95% CI	Without Contract *n* (%)	95% CI	Total *n* (%)	95% CI	χ^2^ (Wald)	*p* Value
Sex								
Male	1986 (61.5%)	59.0–63.8	287 (51.6%)	45.9–57.3	2273 (60.2%)	57.9–62.5	11.49	0.0008
Female	2372 (38.5%)	36.1–40.9	548 (48.4%)	42.7–54.1	2920 (39.8%)	37.5–42.1	-	-
Age group								
18–29 years	1096 (21.0%)	19.3–22.9	278 (33.6%)	28.4–39.2	1374 (22.6%)	20.8–24.6	18.39	<0.001
30–44 years	1788 (44.1%)	41.4–46.8	233 (29.9%)	25.4–34.9	2021 (42.3%)	39.7–44.9	-	-
45–64 years	1360 (32.3%)	29.9–34.9	267 (28.5%)	23.9–33.7	1627 (31.9%)	29.5–34.3	-	-
≥65 years old	114 (2.6%)	1.75–3.77	57 (7.9%)	4.98–12.4	171 (3.3%)	2.4–4.4	-	-
Educational level								
Early education	4 (0.07%)	0.02–0.22	5 (1.17%)	0.25–5.29	9 (0.2%)	0.07–0.64	19.92	<0.001
Primary education	286 (5.86%)	4.77–7.19	144 (13.41%)	10.2–17.4	430 (6.8%)	5.7–8.2	-	-
Secondary education	1869 (36.54%)	33.9–39.3	398 (47.45%)	42.3–52.7	2267 (37.9%)	35.5–40.6	-	-
Technical or vocational education	885 (22.56%)	20.8–24.4	97 (11.29%)	8.47–14.9	982 (21.1%)	19.4–23.0	-	-
Professional or postgraduate education	1314 (34.96%)	32.2–37.8	191 (26.68%)	22.6–31.2	1505 (33.9%)	31.4–36.5	-	-
Self-rated health								
Fair or poor	1134 (24.3%)	22.1–26.6	320 (32.8%)	26.9–39.2	1454 (25.4%)	23.4–27.5	6.97	0.009
Good or very good	3231 (75.7%)	73.4–77.9	511 (67.2%)	60.8–73.1	3742 (74.6%)	72.5–76.6	-	-
Depression (physician-diagnosed)								
No	3594 (84.6%)	82.4–86.5	650 (80.7%)	75.6–84.9	4244 (84.1%)	82.0–86.0	2.46	0.118
Yes	748 (15.5%)	13.5–17.6	179 (19.3%)	15.1–24.4	927 (15.9%)	14.1–18.0	-	-
Diabetes								
No	4076 (92.8%)	91.4–94.0	761 (93.6%)	90.5–95.8	4837 (92.9%)	91.6–94.0	0.32	0.575
Yes	289 (7.2%)	6.0–8.6	70 (6.4%)	4.2–9.5	359 (7.1%)	6.0–8.4	-	-
Hypertension								
No	3866 (88.3%)	86.6–89.8	698 (85.7%)	82.0–88.8	4564 (88.0%)	86.5–89.3	1.78	0.183
Yes	499 (11.7%)	10.2–13.4	133 (14.3%)	11.2–18.0	632 (12.0%)	10.7–13.5	-	-
Overweight or obesity								
No	3274 (74.8%)	72.0–77.5	584 (73.8%)	69.3–77.8	3858 (74.7%)	72.3–77.0	0.14	0.706
Yes	1091 (25.2%)	22.5–28.0	247 (26.2%)	22.2–30.7	1338 (25.3%)	23.0–27.8	-	-
Quality of life								
Good	3532 (81.5%)	79.4–83.5	583 (72.0%)	66.2–77.2	4115 (80.3%)	78.3–82.1	12.69	<0.001
Poor	833 (18.5%)	16.5–20.6	248 (28.0%)	22.8–33.8	1081 (19.7%)	17.9–21.7	-	-
Musculoskeletal disorders								
No	3759 (86.4%)	84.5–88.1	684 (84.8%)	80.4–88.3	4443 (86.2%)	84.5–87.7	0.61	0.436
Yes	606 (13.6%)	11.9–15.5	147 (15.2%)	11.7–19.6	753 (13.8%)	12.3–15.5	-	-
Infertility								
No	4290 (98.5%)	98.0–98.9	819 (98.7%)	96.9–99.4	5109 (98.5%)	98.1–98.9	0.04	0.847
Yes	75 (1.5%)	1.1–2.0	12 (1.3%)	0.6–3.1	87 (1.5%)	1.1–1.9	-	-
Hepatitis B								
No	4332 (99.3%)	98.8–99.6	827 (98.8%)	95.0–99.7	5159 (99.3%)	98.8–99.6	0.58	0.446
Yes	33 (0.7%)	0.4–1.2	4 (1.2%)	0.3–5.0	37 (0.7%)	0.4–1.2	-	-

Note: Counts (*n*) correspond to the unweighted sample (*n* = 5193). Percentages (%) are weighted estimates based on the ENSEX 2022–2023 expansion weights and the complex survey design (strata, primary sampling units, and calibrated weights). Associations were assessed using design-based Wald tests, reported as χ^2^ statistics with one degree of freedom and the corresponding *p* values.

**Table 2:** Association between absence of a labor contract and health indicators (crude and adjusted models).

Health Indicator	Crude OR (95% CI)	*p* Value	Adjusted OR (95% CI)	*p* Value
Self-rated general health	0.66 (0.48–0.90)	0.009	0.78 (0.56–1.08)	0.139
Depression (physician-diagnosed)	1.31 (0.93–1.84)	0.119	1.22 (0.86–1.74)	0.267
Anxiety and depressive symptoms (PHQ-4)	1.62 (1.28–2.07)	<0.001	1.59 (1.26–2.01)	<0.001
Diabetes	0.87 (0.54–1.40)	0.575	0.62 (0.32–1.17)	0.138
Hypertension	1.26 (0.90–1.76)	0.184	1.00 (0.61–1.63)	0.996
Overweight or obesity	1.05 (0.80–1.40)	0.707	1.07 (0.82–1.41)	0.616
Quality of life	0.58 (0.43–0.79)	<0.001	0.71 (0.51–0.99)	0.042
Musculoskeletal disorders	1.14 (0.82–1.60)	0.437	1.00 (0.72–1.40)	0.989
Infertility	0.91 (0.34–2.42)	0.847	0.88 (0.34–2.24)	0.781
Hepatitis B	1.82 (0.38–8.74)	0.453	1.97 (0.36–10.79)	0.432

## Data Availability

The data supporting this study are openly accessible at https://datos.gob.cl/dataset/encuesta-nacional-de-salud-sexualidad-y-genero-enssex-2022-2023/resource/33d3c338-ac5a-46eb-a46c-82c8dedfcc2d?inner_span=True (accessed on 16 October 2025).
